# The return of Lamarck?

**DOI:** 10.3389/fgene.2013.00010

**Published:** 2013-02-05

**Authors:** Etienne Bucher

**Affiliations:** Botanical Institute, University of BaselBasel, Switzerland

In the last decade researchers have produced immense amounts of genetic data resulting in many sequenced genomes. Although we may now know the DNA sequence in a genome, just as seeing all the letters of a book, deciphering which sequences encode genes, let alone understanding which combination of genes are required to organize life, is like decoding the meaning of a book written in a language never before seen. Moreover, how does a cell know which genes to turn on and which to turn off? How does a cell control differentiation into various tissues? For this a cell has to know where it comes from (memory) and what to do (encoded by the DNA). So, how does cellular memory exist and how is it transmitted to the next cell after each cell division? What happens if there is a failure in the transmission of that memory? Can such a memory even be transmitted to the next generation of an organism? This last question is very important since it raises the question as to whether our lifestyle affects our children and grand-children. All these questions are currently being addressed in the research field called “epigenetics.” In recent years, epigenetics has rapidly expanded into all biological disciplines but with numerous, different definitions of what epigenetics is. Therefore, it is not easy for students and advanced researchers to find a good entry point in this complex, albeit fascinating subject.

This is where the book by Olga and Igor Kovalchuk comes into play. It is one of the first books to broadly discuss all the different aspects and mechanisms of epigenetics. The book begins with a historical perspective starting in the beginning of the nineteenth century that proceeds to today providing an overview of how the ideas about epigenetics have evolved, as well as addressing issues that remain controversial. The historical perspective focuses on theories first proposed by Jean-Baptiste Lamarck, whose ideas Charles Darwin did not completely reject, providing a good assessment of how scientists were thinking and debating at that time (Read a free excerpt from the book: http://www.ftpress.com/articles/article.aspx?p=1896675). Next, the authors discuss the molecular pathways involved in epigenetics. In these chapters the authors provide mechanistic details along with excellent graphics to discuss the most recent aspects of chromatin remodeling, DNA methylation, histone modifications and non-coding RNAs. However, the book does not discuss less obvious examples considered as “epigenetic” phenomena by some researchers, such as modifications caused by prions.

After describing epigenetics at the molecular level, the authors explain transgenerational effects mediated by epigenetic mechanisms and different health related subjects, such as the failure of proper transmission of epigenetic memory being associated with cancer and the interesting roles of epigenetics within the context of neuroscience. This chapter connects epigenetics and health with a focus on diet and toxicology, or stated more directly: our lifestyle. Here they summarize how our environment and diet directly impinge on our epigenome, implying that we may not only transmit altered epigenomes affecting the next generation, but that altered epigenomes may more immediately impact our own health. To discuss these aspects the book covers several examples of inheritance of stress memory across generations. The last chapter of the book covers technical applications of RNA silencing [or RNA interference (RNAi)], a very efficient method to silence gene expression by providing small interfering RNAs homologous to the gene to be silenced, which is useful if one cannot just remove a gene.

Interestingly, although the book's title indicates a primary focus on the role of epigenetics in health and disease, which is indeed covered, an underlying strength is the broad discussion covering the different roles of epigenetics in numerous organisms, including plants. This approach benefits the reader by providing the background relevant to the actual discovery and the major developments in the field. This approach reflects the expertise and competencies of the authors, both of whom are medical doctors and currently perform basic plant and cancer research. While the title of the book could have been more general to attract a broader audience, it is a great read for anyone interested in epigenetics. A very useful resource for readers involved in teaching are the “Exercises and Discussion Topics” section at the end of each chapter. This allows the reader to reflect on the content and is a good starting point for exercises with students. Also, information boxes give further details and definitions about selected topics. This book is a must for any library located in institutions involved in molecular biology.

So, is Lamarck now returning to favor? Perhaps his theories regarding the “inheritance of acquired characteristics” might now indeed be explained by epigenetic mechanisms. However, Lamarck saw this as a sole method of transmitting information from one generation to another, which remains in contrast to the inheritance of genetic (hard inheritance) and epigenetic (soft inheritance) information between generations. Thus, Lamarck's main contribution, aside of his concepts of biological evolution, remain limited to being the first to formulate the idea that “transmission of influential circumstances of life” to the progeny can occur. A very interesting question to address in the future will be to determine if the transition from soft to hard inheritance actually takes place.

The book can be found here: http://www.ftpress.com/store/epigenetics-in-health-and-disease-9780132789998

